# Fewer tumour-specific PD-1^+^CD8^+^ TILs in high-risk “Infiltrating” HPV^−^ HNSCC

**DOI:** 10.1038/s41416-020-0966-8

**Published:** 2020-07-03

**Authors:** Ke Xu, You Fu, Yong Han, Ronghui Xia, Shengming Xu, Shengzhong Duan, Zhiyuan Zhang, Jiang Li

**Affiliations:** 1 Shanghai Key Laboratory of Stomatology, Shanghai, P.R. China; 2 Department of Oral and Maxillofacial-Head Neck Oncology, Ninth People’s Hospital, Shanghai Jiao Tong University School of Medicine, Shanghai, P.R. China; 3 Department of Oral Pathology, Ninth People’s Hospital, Shanghai Jiao Tong University School of Medicine, Shanghai, P.R. China; 4 Laboratory of Oral Microbiology, Shanghai Research Institute of Stomatology, Ninth People’s Hospital, Shanghai Jiao Tong University School of Medicine, Shanghai, P.R. China

**Keywords:** Tumour immunology, Head and neck cancer

## Abstract

**Background:**

The prognosis of HPV^-^ HNSCC was worse than that of HPV^+^ HNSCC. Analysis of tumours and tumour-infiltrating lymphocytes (TILs) may provide insight into the progression of HPV^−^ HNSCC.

**Methods:**

The tumour and TIL phenotypic characteristics of 134 HNSCC specimens (HPV^−^ tumours were classified into “Infiltrating” and “Pushing” subtypes based on their different tumour nest configuration and prognosis) were retrospectively analysed. HNSCC data from the Cancer Genome Atlas (*n* = 263) were analysed for CD8α, HPV and overall survival (OS). A murine HNSCC model was used to verify the antitumour role of PD-1^+^CD8^+^ TILs.

**Results:**

The “Infiltrating” HPV^−^ subtype showed shorter OS than the “Pushing” subtype. Moreover, there is a tendency from “Pushing” to “Infiltrating” subtype from the primary to the recurrent lesion. Different from total CD8^+^ TILs, tumour-specific PD-1^+^CD8^+^ TILs were fewer in invasive margin (IM) of “Infiltrating” HPV^−^ tumours. PD-1^+^CD8^+^ TILs recognised autologous HNSCC cells and showed stronger inhibition of tumour growth in a murine HNSCC model resistant to PD-1 blockade.

**Conclusions:**

Coevolution of HPV^−^ HNSCC and TILs is characterised by an “Infiltrating” phenotype and less tumour-specific PD-1^+^CD8^+^ TILs, which may provide a framework for further translational studies and patient stratification.

## Background

Head and neck squamous cell carcinoma (HNSCC) is a heterogeneous group of malignant tumours that include human papillomavirus-positive (HPV^+^) and HPV-negative (HPV^−^) tumours with different prognosis.^1–3^ Several immune-checkpoint receptor inhibitors (ICIs) were approved by FDA for the treatment of HNSCC, but the objective response rate is low.^4–6^ Compared with HPV^+^ HNSCC, HPV^−^ HNSCC has a much worse prognosis, but the roles of TILs in the progression of HPV^−^ HNSCC remain elusive.^7–9^ The coevolution between HNSCC and immune microenvironment accounts for the carcinogenesis and PD-1/PD-L1-blockade resistance of HNSCC.^10,11^ Given that HPV^−^ and HPV^+^ HNSCC differ in terms of aetiology and prognosis, independent analysis of HPV^−^ HNSCC could potentially advance our knowledge of the mechanisms underlying their pathogenesis and immune evasion.

The HPV^−^ HNSCC were further divided into “Pushing” and “Infiltrating” types in this study according to different characteristics of tumour nest in IM and different prognosis.^12–16^ Although the grading of invasive margin of HNSCC has high prognostic value, the relationship between different subtypes and the immune subsets in tumour microenvironment has not been elucidated.

Our hypothesis is that HPV^−^ HNSCC coevolves with CD8^+^ T cells, and tumour-specific CD8^+^ T cells may play a pivotal role in the process. PD-1^+^CD8^+^ TILs were rich in tumour-specific cytotoxic T cells, but their distribution and alterations during the progression of HNSCC progression remain unclear.^17,18^ Understanding the evolutionary stage of a particular tumour may be a necessary step in designing personalised treatment strategies for each patient in the next generation of immunotherapy. In this study, unbiased whole-tumour section analysis combined with flow cytometry showed that tumour-specific PD-1^+^CD8^+^ TILs not only showed greater tumour exposure, but also had higher prognostic value for HNSCC than PD-1^−^CD8^+^ TILs. Moreover, using a syngeneic HNSCC model resistant to PD-1 blockade, the results indicated that PD-1^+^CD8^+^ TILs could still recognise autologous tumour cells and inhibit tumour growth. It suggested that PD-1^+^CD8^+^ TILs, rather than simply exhausted T cells, play a key antitumour role in the coevolution between HPV^−^ HNSCC and the immune microenvironment. These results may provide insight into the coevolution of HPV^−^ HNSCC and tumour-specific PD-1^+^CD8^+^ TILs.

## Methods

### Patients and specimens

Tumour samples of patients with HNSCC were obtained between 2007 and 2017. For HPV detection of primary HNSCC, p16 immunohistochemistry (over 70% of tumour cells staining positive for p16 were considered as p16^+^) as a surrogate marker, and HPV genotyping test (23 types, Yaneng Biosciences) was done for all oropharyngeal tumours. All primary or recurrent tumours from the tonsils or lymph nodes were excluded, considering that surrounding lymphocytes in these subsites may interfere with the phenotypic analysis. Ultimately, 101 primary tumour samples, 22 matched local recurrent HPV^−^ samples and 11 primary HPV^+^ samples from the same centre were obtained. Histologic examination was performed by two pathologists. Tumour grade, stage and basic patient clinical information of HPV^−^ primary tumour samples are summarised in Supplementary Table S1.

### Image acquisition and analysis

For IHC analysis, CD8^+^ TILs were evaluated by examining at least ten representative fields (40×) using Fiji software. For immunofluorescence-stained image acquisition, slides were scanned using the Vectra platform (PerkinElmer) or TissueFAXS slide scanning system (TissueGnostics). Analysis was performed using InForm software (PerkinElmer), TissueQuest or StrataQuest software (TissueGnostics). Cells were identified based on segmentation of DAPI-stained nuclei. When StrataQuest software was used, whole-tumour section was scanned, and cells in indicated areas were analysed.

### Animal experiments

Female C3H/He mice aged 6–8 weeks were purchased from the Charles River Laboratories. All mice were maintained under specific pathogen-free conditions in the animal facilities of the Ninth People’s Hospital. Prior to treatment, mice were randomised. Nasal anaesthesia (isoflurane) was used before the subcutaneous injection. SCC7 cells were implanted by subcutaneous injection of 2 × 10^5^ cells into the right flanks. α-PD-1 (200 μg) and IgG isotype (200 μg) were administered intraperitoneally twice weekly. In total, 3 × 10^6^ PD-1^+^CD8^+^ or PD-1^−^CD8^+^ TILs from autologous tumours were administered intravenously on day 4 after the inoculation. Tumour size was calculated as length × width. Nasal anaesthesia (isoflurane) was used before the tumours were collected, and the mice were then killed by cervical dislocation. Mice with length greater than 2 cm were euthanized (using CO_2_ inhalation) for ethical consideration.

### Statistical methods

For statistical analysis, first, the normal distribution of the data was analysed using Kolmogorov–Smirnov test. If normal distribution was assumed, the parametric test was used (unpaired *t* test for pairwise comparison or ordinary one-way ANOVA for multiple comparisons); if not, the nonparametric test was used (Mann–Whitney test for pairwise comparison or Kruskal–Wallis test for multiple comparisons). All multiple comparisons were corrected by Bonferroni test. Overall survival (OS) was defined as the time from initial diagnosis until death or the most recent follow-up. Survival distributions were displayed using the Kaplan–Meier method, and their comparisons were conducted using Log-rank test, unless otherwise indicated. The results are presented as the mean ± standard deviation (SD). *P* < 0.05 was considered statistically significant.

## Results

### “Infiltrating” is a high-risk HPV–HNSCC subtype

**Fig. 1:**
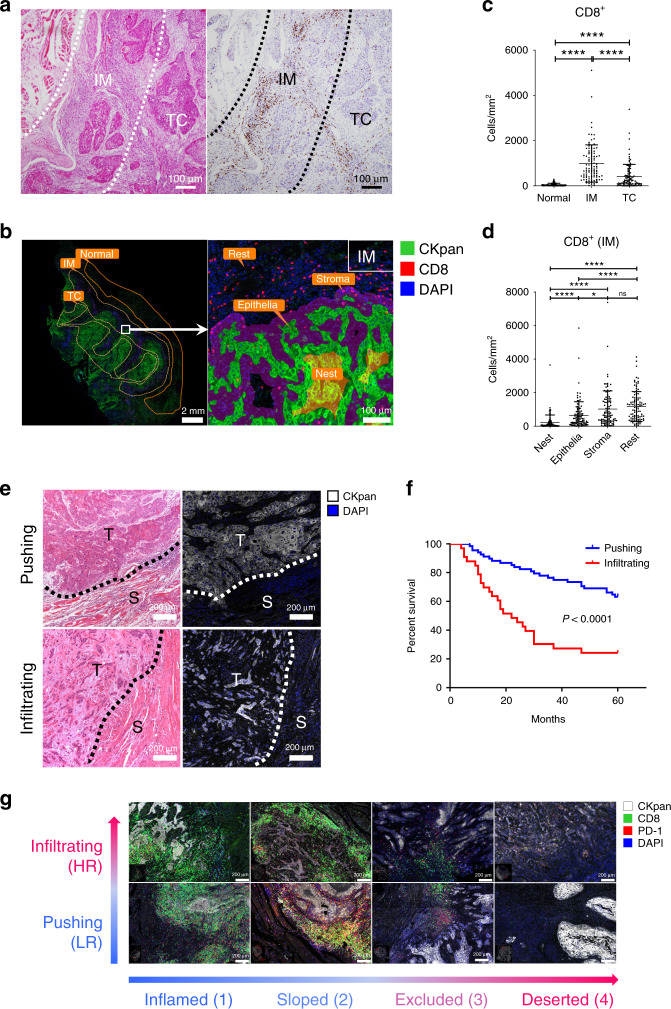
“Infiltrating” is a high-risk HPV^−^ HNSCC subtype. **a** Representative image of CD8^+^ TILs in HPV^–^ HNSCC. Left, haematoxylin and eosin (H&E) staining. Right, IHC staining of CD8^+^ TILs. IM: invasive margin; TC: tumour centre. **b** Representative image of zoning and machine-based CD8^+^ quantification. Multiplex IHC staining of CKpan, CD8 and DAPI. The CKPan^+^ areas (green) were automatically recognised as tumour. Boxes represent the magnified area on the right. IM and TC were further divided into four parts: stroma (from tumour border to 33 μm outside the border, purple overlay), epithelia (from 33 μm inside the tumour border to the border, green overlay), nest (areas other than “epithelia” within the tumour, orange overlay) and rest (areas other than “stroma” within the stroma). Biomarkers and colours are shown on the upper right. **c**, **d** Densities of CD8^+^ in indicated areas analysed by StrataQuest, *n* = 101, data are shown as mean with SD. *P < 0.05; ****P < 0.0001; ns not significant. **e** Representative images of “Pushing” and “Infiltrating” HPV^−^ HNSCC subtype. Dashed line, boundaries between the cancer nest and the cancer stroma. T: tumour; S: stroma. **f** Kaplan–Meier analysis of OS of HNSCC patients classified as indicative subgroups. Log-rank test was performed to determine significance. **g** Representative images of different patterns of HPV^−^ HNSCC based on the tumour shape and CD8^+^ TIL distribution in IM. PD-1 and CD8 staining in eight HPV^−^ HNSCC subtypes, HR stands for “high risk” and LR for “low risk”. Biomarkers and colours are shown on the upper right.

The prognostic value of CD8^+^ TILs for HNSCC remains controversial.^19–21^ Since the prognosis of HPV^-^ HNSCC is worse than that of HPV^+^ HNSCC, we first analysed HPV^−^ HNSCC.^22–24^ In total, 101 formalin-fixed paraffin-embedded (FFPE) primary HNSCC samples were selected by the following criteria: (1) surgical resection was the primary treatment, and no preoperative chemoradiotherapy was performed, (2) HPV status was known and (3) adequate IM and tumour centre (TC) areas were available for analysis. The density of CD8^+^ TILs at IM and TC was quantified (Fig. 1a; Supplementary Fig. S1a). No significant correlation was found between OS and the density of CD8^+^ T cells in different areas (IM, TC or combined) (Supplementary Fig. S1b–e). To minimise selection bias, machine-based cell quantification was performed on the whole section after immunofluorescence staining. Each section was divided into three areas: IM, TC and normal. IM and TC were further divided into four subareas: stroma, epithelia, nest and rest (Fig. 1b). CD8^+^ TILs were densest in the IM (especially in the subarea stroma, or IM_stoma), suggesting a more intense immune response in this region (Fig. 1c–d). However, there was no significant correlation between OS and CD8^+^ TIL densities in different areas/subareas (Supplementary Fig. S1f–i).

To confirm these results in another cohort, the Cancer Genome Atlas (TCGA) samples (*n* = 263) with CD8α expression and HPV status were analysed.^25^ Three groups were divided: HPV^−^CD8^low^ (*n* = 113), HPV^−^CD8^high^ (*n* = 112) and HPV^+^ (*n* = 38) (Supplementary Fig. S2a, b). Although HPV^+^ was associated with higher OS, there was no significant difference in OS between HPV^−^CD8^low^ and HPV^−^CD8^high^ groups (Supplementary Fig. S2c–f). Similarly, no significant correlation was found between OS and the CD8α expression in 38 HPV^+^ patients (Supplementary Fig. S2g). These results suggest that HPV^−^ HNSCC and CD8^+^ TILs may need further stratification to understand the relationship between pre-existing adaptive immune response and prognosis of patients with HPV^−^ HNSCC.

Considering that tumour cells in IM are frequently different from those in other parts, and according to reports on the classification of tumour shape in IM,^12–14^ we divided 101 HPV^−^ HNSCC samples into two subtypes: “Pushing” and “Infiltrating” (Fig. 1e). In “Pushing” subtype, most tumour nests were big oval with smooth borders; in “Infiltrating” subtype, most nests were small, scattered with irregular margins (the percentage of small tumour nests in IM is 0–20% in the “Pushing” and 20–100% in the “Infiltrating” subtype).^13^ To be noticed, there was no significant difference between “Infiltrating” and “Pushing” subtypes in the proportion of poor differentiation or high grade (Supplementary Fig. S3a–c). However, “Infiltrating” HNSCC had statistically significant worse OS than “Pushing” HNSCC (Fig. 1f, *P* < 0.0001).

To analyse the correlation between HNSCC and TILs, we analysed the distribution of CD8^+^ TILs in different HNSCC subtypes. Based on the differences of CD8^+^ TIL patterns, “Infiltrating” and “Pushing” HNSCC were further divided into four subtypes: inflamed, sloped, excluded and deserted (Supplementary Fig S4a; Fig. 1g). It is noteworthy that HNSCC with high CD8^+^ TILs, inflamed and sloped, accounts for 84.1% of this cohort. In IM_stroma (Fig. 1b), the percentage of CD8^+^ TILs increased from “deserted” to “inflamed” (Supplementary Fig. S4b).

To analyse the coevolutionary trend, 22 locally recurrent HNSCC cases that were matched with 18 primary HPV^−^ HNSCC cases were analysed (Supplementary Fig. S4c and Supplementary Table S2). Among primary-to-recurrent lesion transitions, 15 (68.2%) were pattern changes; among 6 tumour changes, 5 (83.3%) were “Pushing” to “Infiltrating” transition; among 11 CD8^+^ TIL pattern changes, all were “inflamed” to “deserted” transition (Supplementary Fig. S4d–f; Fig. 1g). Taken together, the evolution of HPV^−^ HNSCC is characterised by increased invasiveness and immune evasion, and CD8^+^ TILs play a role in the coevolution of HPV^−^ HNSCC and immune microenvironment.

### PD-1^+^CD8^+^ cells constitute the primary PD-1^+^ TILs

**Fig. 2:**
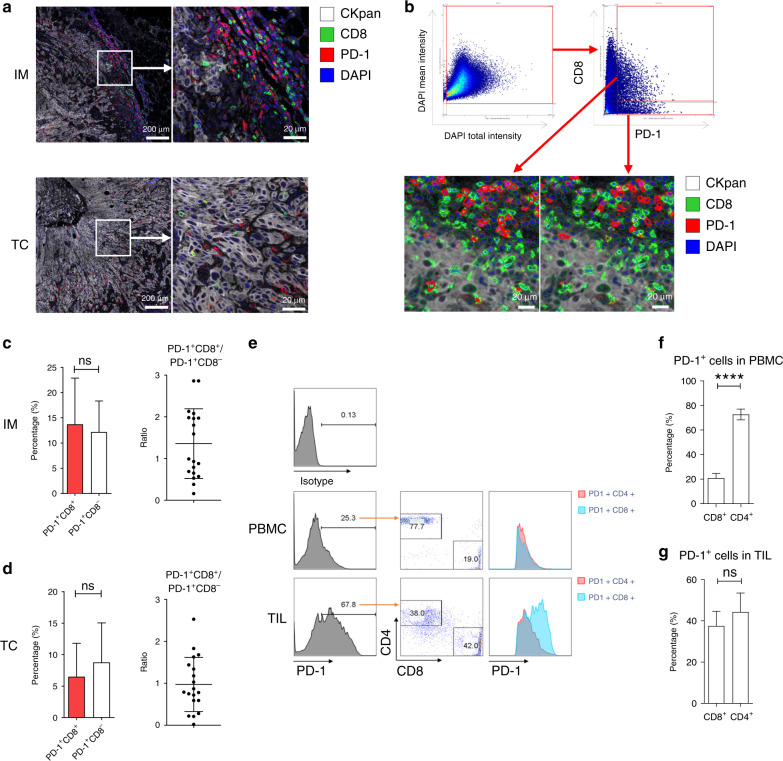
PD-1^+^CD8^+^ cells are a major subset of PD-1^+^ TILs. **a** Representative images of IM and TC of HNSCC. Boxes represent the magnified area on the right. Biomarkers and colours are shown on the upper right. **b** Gating strategy for image cytometry of CD8 and PD-1 using StrataQuest platform. **c**, **d** Percentage of indicated cells and ratio of PD-1^+^CD8^+^ to PD-1^+^CD8^–^ in IM (**c**) or TC (**d**) (*n* = 19; order of antibodies: CD8 and PD-1, analysed by TissueQuest). **e** Flow-cytometry analyses of PBMC and TILs is shown. **f**, **g** Percentage of indicated cells in PBMC (**f**) and TILs (**g**) (n = 5). **c**, **d**, **f** and **g** Data are shown as mean with SD. ***P < 0.001; ns not significant.

To find the key CD8^+^ TIL subgroup in HNSCC immune microenvironment, we first evaluated the frequency of different subsets of PD-1^+^ TILs, which is the basis of PD-1/PD-L1 immunotherapy^26,27^ (Fig. 2a). Image cytometric quantification was performed on “Pushing” and “Infiltrating” HNSCC samples, respectively, and the distribution of PD-1^+^CD8^+^ TILs in different subregions of the two HNSCC subtypes was compared (Fig. 2b). The results showed that PD-1^+^CD8^+^ T cells constituted the primary PD-1^+^ TILs in IM and TC (Fig. 2c, d). Next, matched samples from HPV^−^ HNSCC patients were used to evaluate the frequency of PD-1^+^CD8^+^ and PD-1^+^CD4^+^ cells in peripheral blood and tumour tissues by flow cytometry. In PBMCs, the proportions of PD-1^+^CD4^+^ cells were higher than those of PD-1^+^CD8^+^ cells, but the proportions of the two in tumours were comparable (Fig. 2e–g). The relative enrichment of PD-1^+^CD8^+^ cells in tumours supports the idea that PD-1^+^CD8^+^ TILs are rich in tumour-specific cytotoxic T cells.

### Special distribution pattern of PD-1^+^CD8^+^ in HPV^–^ HNSCC

**Fig. 3:**
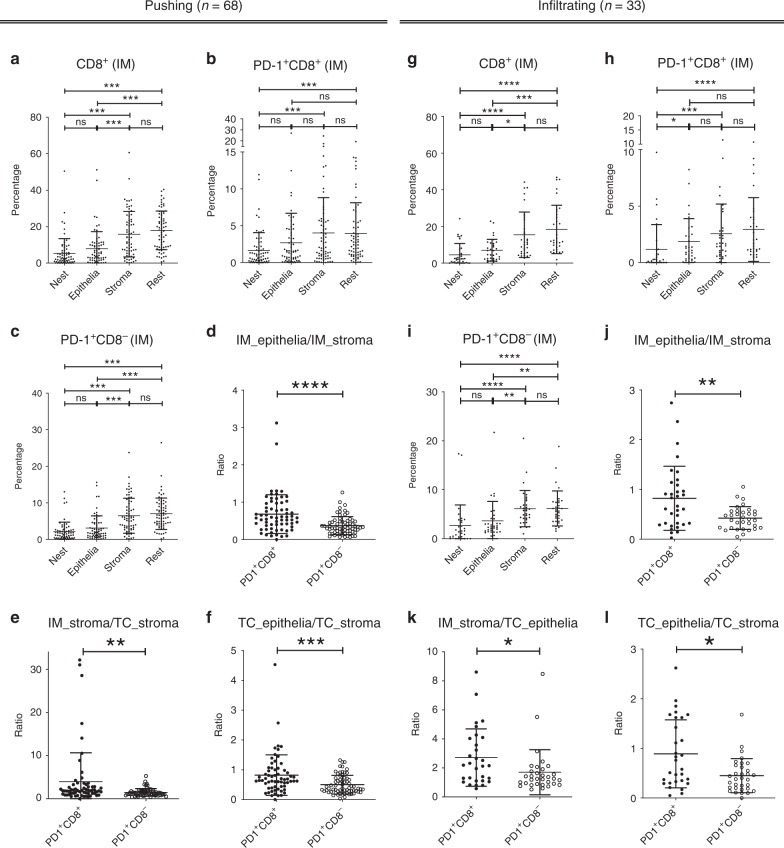
Distribution pattern of PD-1^+^CD8^+^ TILs in HPV^−^ HNSCC. Percentages and ratios of indicated cells in indicated subareas for “Pushing” (**a**–**f**, *n* = 68) or for “Infiltrating” HPV^−^ HNSCC (**g**–**l**, *n* = 33). (**a**–**c** and **g**–**i**) Percentage of indicated cells out of total cells in indicated subareas. **d**–**f**, **j**–**l** Ratio of indicated cells in different subareas: IM_epithelia to IM_stroma (**d**, **j**), IM_stroma to TC_stroma (**e**, **k**) and TC_epithelia to TC_stroma (**f**, **l**). **a**–**l** Data analysed by StrataQuest are shown as mean with SD. *P < 0.05; **P < 0.01; ***P < 0.001; ****P < 0.0001; ns not significant.

To gain insight into the distribution patterns of different TIL subsets, the entire sections of 101 HNSCC samples were analysed. In IM of “Pushing” and “Infiltrating” HPV^–^ HNSCC, the percentages of total CD8^+^, PD-1^+^CD8^+^ and PD-1^+^CD8^−^ TILs decreased from “rest” to “nest” (Fig. 3a–c, g–i). Different from total CD8^+^ or PD-1^+^CD8^−^ TILs, the percentages of PD-1^+^CD8^+^ TILs in IM_stroma and IM_epithelia (subarea epithelia of IM) were similar. In addition, the ratios of IM_epithelia PD-1^+^CD8^+^ TILs to IM_stroma PD-1^+^CD8^+^ TILs were higher than those of PD-1^+^CD8^−^ TILs (Fig. 3d, j). For both HPV^−^ HNSCC subtypes, the ratios of IM_stroma PD-1^+^CD8^+^ TILs to TC_stroma (subarea stroma of TC) PD-1^+^CD8^+^ TILs were higher than those of PD-1^+^CD8^−^ TILs (Fig. 3e, k). Similar to the situation in IM, the PD-1^+^CD8^+^ was more likely to present in the tumour nest of TC (Fig. 3f, l). These results suggested that PD-1^+^CD8^+^ TILs were more likely to interact with HNSCC cells, which also supports the tumour specificity of PD-1^+^CD8^+^ TILs in HNSCC.

### PD-1^+^CD8^+^ TILs were a favourable prognostic biomarker in HNSCC

**Fig. 4:**
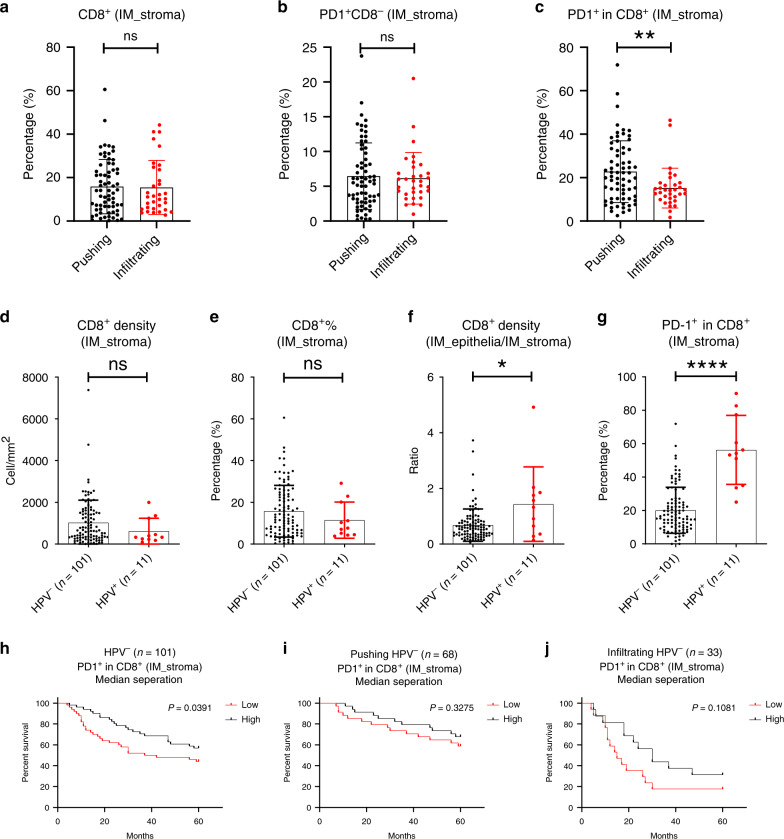
The correlation between the percentage of PD-1^+^CD8^+^ TILs in IM_stroma and the prognosis of different HNSCC subtypes. **a**–**c** Percentages of indicated cells were compared between “Pushing” (*n* = 68) and “Infiltrating” HPV^–^HNSCC (*n* = 33). **d**–**g** Densities, percentages or ratios of indicated cells in indicated subareas. **h**–**j** Kaplan–Meier analysis of OS of HNSCC patients classified as indicative subgroups. Gehan–Breslow–Wilcoxon test was performed to determine significance. **a**–**l** Data analysed by StrataQuest are shown as mean with SD. **P < 0.01; ***P < 0.001; ****P < 0.0001; ns not significant. IM_stroma, subarea stromal of IM; IM_epithelia, subarea epithelia of IM; TC_stroma, subarea stromal of TC; TC_epithelia, subarea epithelia of IM.

Different from total CD8^+^ TILs and PD-1^+^CD8^−^ TILs, the percentages of PD-1^+^CD8^+^ TILs in IM_stroma of “Infiltrating” subtype were lower than those in “Pushing” HNSCC (Fig. 4a–c). PD-1^+^CD8^+^ TILs also decreased in IM_epithelia, while the trend in IM_rest was relatively insignificant (Supplementary Fig. S5a–f). Since many ICI-based immunotherapies rely on the tumour-specific PD-1^+^CD8^+^ TILs, their lacking in high-risk “Infiltrating” HPV^−^ HNSCC may exacerbate the resistance of these HNSCCs to immunotherapy.

To further understand the differences in TILs between the two HPV^−^ HNSCC subtypes, the percentages of B cells (CD20^+^), Tregs (FOXP3^+^), NK cells (CD56^+^) and macrophages (CD68^+^) were analysed. There were little NK cells (Supplementary Fig. S6 a, b). There was no significant difference in the densities of CD68^+^ TAMs and Tregs between the two subtypes (Supplementary Fig. S6c, d). B cells were fewer in IM of “Infiltrating” HPV^−^ HNSCC (Supplementary Fig. S6e). The PD-L1^+^CD68^+^ macrophages were surrounded by PD-1^+^CD8^+^ TILs in most cases, suggesting that they were pivotal in the negative regulation of PD-1^+^CD8^+^ TILs (Supplementary Fig. S7).

To better understand the association between PD-1^+^CD8^+^ TILs and HNSCC, the PD-1^+^CD8^+^ TILs between HPV^−^ HNSCC (*n* = 101) and HPV^+^ HNSCC (*n* = 11) were analysed (Supplementary Table S3). The results showed that the density and percentage of CD8^+^ TILs were comparable in “IM_stroma” of HPV^+^ HNSCC and HPV^−^ HNSCC, but CD8^+^ T cells were more prone to interact with tumour cells in HPV^+^ HNSCC (Fig. 4d–f). Moreover, the percentages of PD-1^+^CD8^+^ TILs in IM_stroma of HPV^+^ HNSCC were higher than those in HPV^−^ HNSCC (Fig. 4g). Kaplan–Meier analysis was used to evaluate OS and PD-1^+^CD8^+^ percentages; the data suggested that HPV^−^ HNSCC with lower percentage of PD-1^+^CD8^+^ cells in IM_stroma had worse OS than that with higher percentage of PD-1^+^CD8^+^ cells in IM_stroma (Fig. 4h–j).

In summary, these results suggested that PD-1^+^CD8^+^ TILs have higher prognostic value than other CD8^+^ TILs in HNSCC.

### PD-1^+^CD8^+^ TILs recognise autologous HNSCC cell

**Fig. 5:**
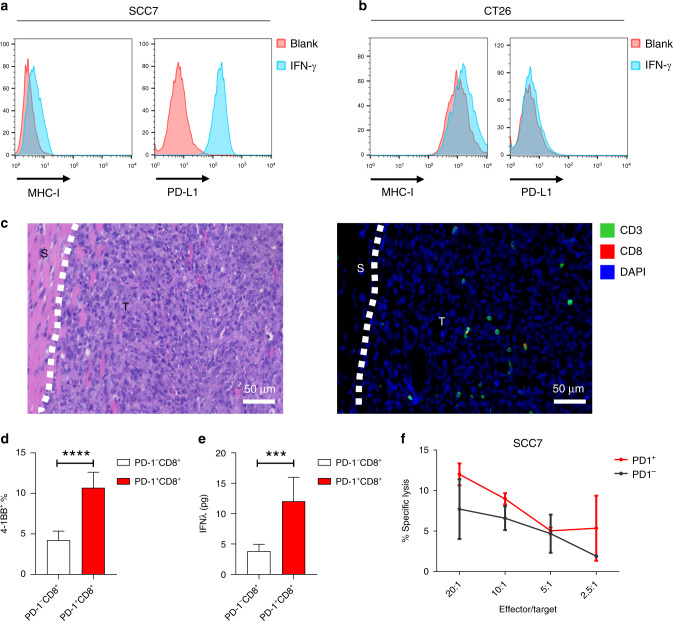
PD-1^+^CD8^+^ TILs, but not PD-1^−^CD8^+^ counterparts, recognise autologous tumour cells in vitro. **a**, **b** Surface expression of MHC-I or PD-L1 on SCC7 and CT26 with or without IFN-γ stimulated. **c** Representative images of IM region of SCC7 tumour. Left, H&E staining; right, multiplex IHC staining; Dashed line, boundaries between the cancer nest and the cancer stoma. T: tumour; S: stroma. Biomarkers and colours are shown on the upper right. **d**, **e** Response of SCC7-derived TILs to SCC7. Indicated CD8^+^ TILs were cocultured with SCC7 for 24 h. Tumour recognition was assessed by measuring the frequency of CD8^+^4-1BB^+^ cells (**c**) and IFN-γ release (**f**). Cytotoxicity of SCC7 by indicated CD8^+^ TILs. **d**, **e** Data are shown as mean with SD. ***P < 0.001; ****P < 0.0001; ns not significant.

To verify the antitumour function of PD-1^+^CD8^+^ TILs in HNSCC, SCC7, a head and neck squamous cell carcinoma was used.^28^ The surface expression of MHC-I and PD-L1 of SCC7 cells was elevated in response to IFN-γ (Fig. 5a). However, compared with CT26 cancer cell, the baseline expression of MHC-I of SCC7 was lower, and it showed a significant increase in PD-L1 expression in response to IFN-γ (Fig. 5b). MHC-I is a key component of the antigen-processing and presentation machinery (APM), and its formation defect is an important mechanism of immune evasion and immunotherapy resistance.^29^ Moreover, there were few CD3^+^ and CD8^+^ TILs in SCC7 tumour (Fig. 5c). These results suggested that SCC7 may be resistant to PD-1 blockade.

To investigate the tumour reactivity of PD-1^+^CD8^+^ TILs, we isolated PD-1^+^CD8^+^ TILs and PD-1^−^CD8^+^ TILs from SCC7 tumours, expanded them in vitro and tested their ability to recognise SCC7 cells. After coculture with SCC7 cells, PD-1^+^CD8^+^ TILs had higher 4-1BB upregulation and IFN-γ secretion than PD-1^−^CD8^+^ TILs (Fig. 5d, e). The capability of PD-1^+^CD8^+^ TILs in SCC7 cell lysis was only marginally higher than PD-1^−^CD8^+^ TILs, which might be partly due to low expression of MHC-I in SCC7 (Fig. 5f).

### Autologous PD-1^+^CD8^+^ TIL transfer impairs tumour growth in SCC7 model

**Fig. 6:**
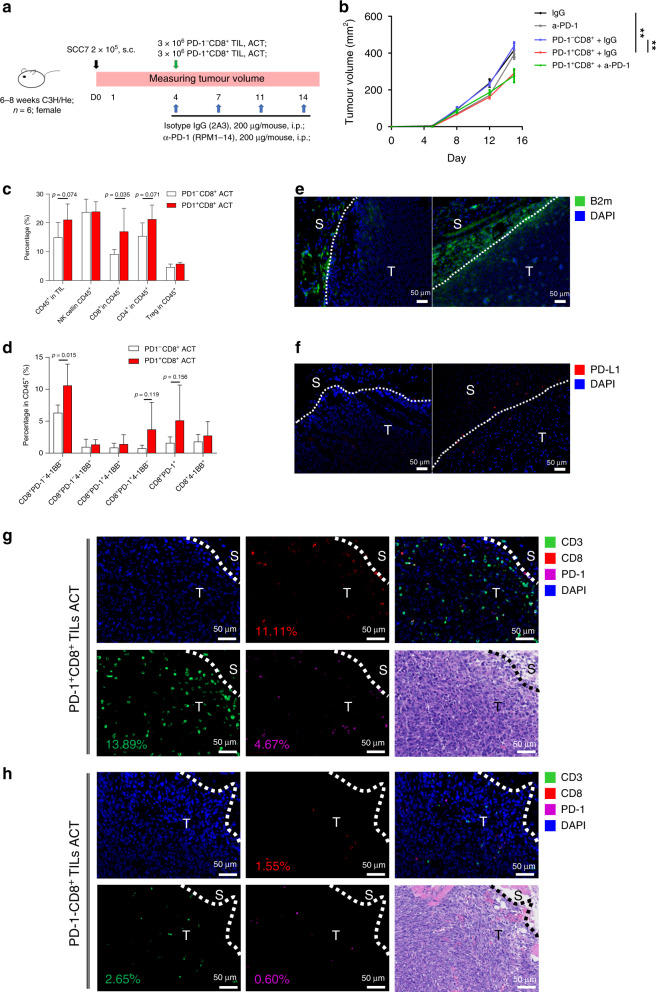
PD-1^+^CD8^+^ TILs inhibit the tumour growth of PD-1-resistant SCC7. **a** Data illustrate treatment regimen. **b** Mean tumour growth. **c**, **d** TILs from tumours of PD-1^–^CD8^+^ TILs ACT and PD-1^+^CD8^+^ TIL ACT group were harvested at day 14 (*n* = 6). **c** CD45^+^ leukocyte and indicated TILs as a percentage of total CD45^+^ were shown. **d** Frequency of 4-1BB and PD-1 expression on CD8^+^ TILs. **e**, **f** Representative images of B2m^+^ and PD-L1^+^ cells in SCC7’s IM region from different treatment groups (left, PD-1^−^CD8^+^ TIL ACT group; right, PD-1^+^CD8^+^ TIL ACT group). **g**, **h** Representative images of CD3^+^, CD8^+^ and PD-1^+^ cells in SCC7’s IM region from PD-1^+^CD8^+^ TIL ACT group (**g**) and PD-1^−^CD8^+^ TIL ACT group (**h**). Dashed line, boundaries between the cancer nest and the cancer stoma. T: tumour; S: stroma. Biomarkers and colours are shown on the upper right. **c**, **d** Data are shown as mean with SD.

Given that PD-1^+^CD8^+^ TILs could recognise SCC7, we hypothesised that the adoptive cell transfer (ACT) of this subset may enhance antitumour immunity (Fig. 6a). As expected, SCC7 was resistant to α-PD-1 treatment. Strikingly, we found that PD-1^+^CD8^+^ TILs ACT, rather than PD-1^−^CD8^+^ TILs ACT, generated significant antitumour activity (Fig. 6b).

To better verify the mechanism of action, changes of SCC7 tumour microenvironment were analysed by flow cytometry. Compared with the PD-1^−^CD8^+^ TIL ACT, PD-1^+^CD8^+^ TIL ACT altered the TIL composition in SCC7 tumours: the percentage of CD8^+^ TILs was increased, and PD-1^+^CD8^+^ TILs also increased slightly in PD-1^+^CD8^+^ TIL ACT group (Fig. 6c, d). Moreover, it was noticed that the immune response of tumour IM was higher in PD-1^+^CD8^+^ TIL ACT group than in the control group: B2m, a component of MHC-I molecules, was elevated in the margin of tumour nest, and there were more PD-L1^+^ TILs (Fig. 6e, f). In addition, the percentage of CD3, CD8 and PD-1^+^CD8^+^ TILs was higher in IM of PD-1^+^CD8^+^ TIL ACT group than that of PD-1^−^CD8^+^ TIL ACT group (Fig. 6g, h).

Taken together, PD-1^+^CD8^+^ TILs more accurately represent the tumour-specific subset than PD-1^−^CD8^+^ TIL counterpart, and hold greater potential for immunotherapy, even for HNSCC with primary resistance to α-PD-1 therapy.

### Functional PD-1^+^CD8^+^ TILs in “Infiltrating” HPV^−^ HNSCC

To know whether the PD-1^+^CD8^+^ TILs in high-risk “Infiltrating” HNSCC subset were mostly exhausted or dysfunctional, we further analysed the HPV^−^ HNSCC primary tumour samples. “Pushing” tumours (*n* = 26) and “Infiltrating” tumours (*n* = 16) that had plenty PD-1^+^CD8^+^ TILs in IM were analysed for CD8, PD-1, TIM3, Granzyme-B (GZMB) and Ki67. The results showed that the percentage of exhausted CD8^+^ TILs (PD-1^+^TIM3^+^) in IM was similar in “Pushing” and “Infiltrating” subsets (Supplementary Fig. S8a). GZMB^+^PD-1^+^CD8^+^ TILs and Ki67^+^PD-1^+^CD8^+^ TILs in “Infiltrating” HNSCC were comparable to those in “Pushing” HNSCC, suggesting that in the “Infiltrating” subset, tumour-specific PD-1^+^CD8^+^ TILs maintained the ability to secrete tumour-killing cytokine and to proliferate in the tumour-immune interacting zone (Supplementary Fig. S8b). The functionality of PD-1^+^CD8^+^ TILs in the “Infiltrating” subset was also supported by the fact that the percentage of GZMB^+^PD-1^+^CD8^+^ TILs and Ki67^+^PD-1^+^CD8^+^ TILs was no less than that in PD-1^−^CD8^+^ TILs (Supplementary Fig. S8c, d).

Taken together, tumour-specific PD-1^+^CD8^+^ TILs play a key antitumour role in the coevolution between HNSCC and the immune microenvironment. However, the decrease in PD-1^+^CD8^+^ TILs should be brought to the forefront in order to help identify high-risk HNSCC patients and give appropriate personalised immunotherapy.

## Discussion

Even though the detailed mechanisms remain unclear, it is widely accepted that tumour evolution is shaped by two major forces: genetic/epigenetic changes of tumour cells and selective pressure from the tumour microenvironment. Immune predation is a major form of selective pressure.^11^ The prognostic effects of CD8^+^ TIL density in HPV^−^ HNSCC remain controversial.^30^ Feng et al. reported that CD8^+^ TILs at the IM correlated with higher OS of patients with HPV^−^ oral squamous cell cancer.^3^ We have also found that CD8^+^ TIL was densest in the IM region; however, either in our cohort or in TCGA samples, there was no correlation between CD8^+^ TIL density and OS in HPV^−^ HNSCC (measured at different cutoffs). There are several possible explanations for this contradiction: (1) small sample size, (2) HPV^−^ HNSCC represents a group of tumours that require further classification and (3) compared with the total CD8^+^ TILs, the PD-1^+^CD8^+^ TILs may more accurately represent the tumour-specific subset and be of greater value to prognosis and immunotherapy.^31,32^

Clinical trials targeting ICR have shown promising results for multiple cancers, and many biomarkers predict the response to PD-1-based immunotherapy, such as PD-L1 expression, tumour mutational burden (also known as mutation load) and CD8^+^ T-cell infiltrates.^33–35^ However, there is still an urgent need for better biomarkers or combinations that improve the response rate of immune-checkpoint therapy in different cancers, including HPV^−^ HNSCC. In this study, we performed additional analyses in the context of HPV^−^ HNSCC to understand the importance of considering tumoral and immune factors as a whole, to gain insights into their primary resistance to immunotherapy as well as to help clinicians optimise personal therapy for cancer patients.

In this study, we performed a proof-of-concept study in the context of HNSCC to understand the importance of considering tumoral and immune factors as a whole. Pathological features of tumour are important for two reasons: firstly, different genotypes may produce the same phenotype; secondly, phenotypic analysis of whole-tumour section, combined with multipoint sequencing, will lead to more accurate tumour typing. The terms “Pushing” and “Infiltrating” used in this study are similar to “Type A” and “Type B” proposed by Nakanishi et al.,^13^ and we proposed “Pushing” and “Infiltrating” only for the convenience of classification and understanding. Our data showed that “Infiltrating” subtype was associated with worse prognosis. From primary to local recurrent tumours, the coevolutionary trend supports the notion that adaptive immune selection is a main selective pressure for HNSCC cells. The phenotypic coevolution of HPV^−^ HNSCC and CD8^+^ TILs may serve as a primary blueprint for patient stratification.

Despite the presence of programmed death-ligand 1 (PD-L1) in >50% HNSCC patients, only a minority of patients with HNSCC (< 20%) respond to ICI immunotherapy.^17,36^ From the perspective of tumour, the HNSCCs may have adopted different types of immune-escape strategies. From the perspective of TILs, CD8^+^ TILs functionally can be further divided into tumour-specific and non-specific groups of cells. This study suggests that the combined analysis of tumours and TILs may help to further increase the response rate of ICI therapy by providing the basis for patient stratification.

HNSCC covers a lot of different subsites, and the risk factors for HNSCC at different subsites are similar: about 80% of HNSCC attributed to tobacco exposures, other risk factors include alcohol, betel nuts, foods high in nitroso compounds and HPV exposure.^37–39^ A potential drawback of this study is that the number of tumours is limited, except for HNSCC in the oral or oropharynx, although oral and oropharyngeal squamous cell carcinoma is the most common type of head and neck carcinomas. This is partly because all primary or recurrent tumours from the tonsils or lymph nodes were excluded, given that lymphocytes around these subsites may interfere with phenotypic analysis. The HPV^−^ HNSCCs were divided into “Pushing” and “Infiltrating” subtypes in this study, but their clinical relevance and definite marker (based on blood or biopsy) need further study. However, the relatively low levels of B cells and PD-1^+^CD8^+^ cells in high-risk “Infiltrating” HPV^−^ HNSCC suggest that ICI therapy may show a higher response if combined with treatments that reverse the lack of PD-1^+^CD8^+^ TILs in these patients. Such treatments are included but not limited to TGF-β blockade, immune modulation, macrophage inhibition or cancer vaccines.^40^

The abundance of PD-1^+^CD8^+^ TILs in most HNSCC patients, 84.1% of the HPV^−^ HNSCC microenvironments were rich in CD8^+^ TILs in this study, provides the basis for immunotherapy. First, PD-1^+^CD8^+^ TIL ACT is a new option with clinical potential for patients with α-PD-1 resistance. Second, since PD-1^+^CD8^+^ TILs are rich in tumour-specific cells, their TCRs can be obtained by single-cell sequencing and bioinformatics analysis, and new personalised TCR weapons may thus be obtained. Moreover, the results suggest that, by combined analysis of HNSCC and PD-1^+^CD8^+^ TILs, we may more accurately understand the immunotyping and escape strategy of the tumour. In summary, through the combined analysis of tumour and tumour-specific CD8^+^ TILs, we could understand the evolutionary status of a tumour more accurately and provide the basis for clinicians to develop personalised treatment plans.

## Supplementary information

Supplementary Materials

## Data Availability

All data are published within this paper and within accompanying supporting files (indicated in text) and accessed via weblink on the journal site.
