# Supplementary material for: Fewer tumour-specific PD-1+CD8+ TILs in high-risk “Infiltrating” HPV− HNSCC
Source: Br J Cancer. 2020 Jul 3;123(6):932–41. doi: 10.1038/s41416-020-0966-8 (PMC7492364; doi:10.1038/s41416-020-0966-8)
Supplement: Supplementary file 1 — Supplementary Materials [file 41416_2020_966_MOESM1_ESM.pdf]

Supplementary Figure S1

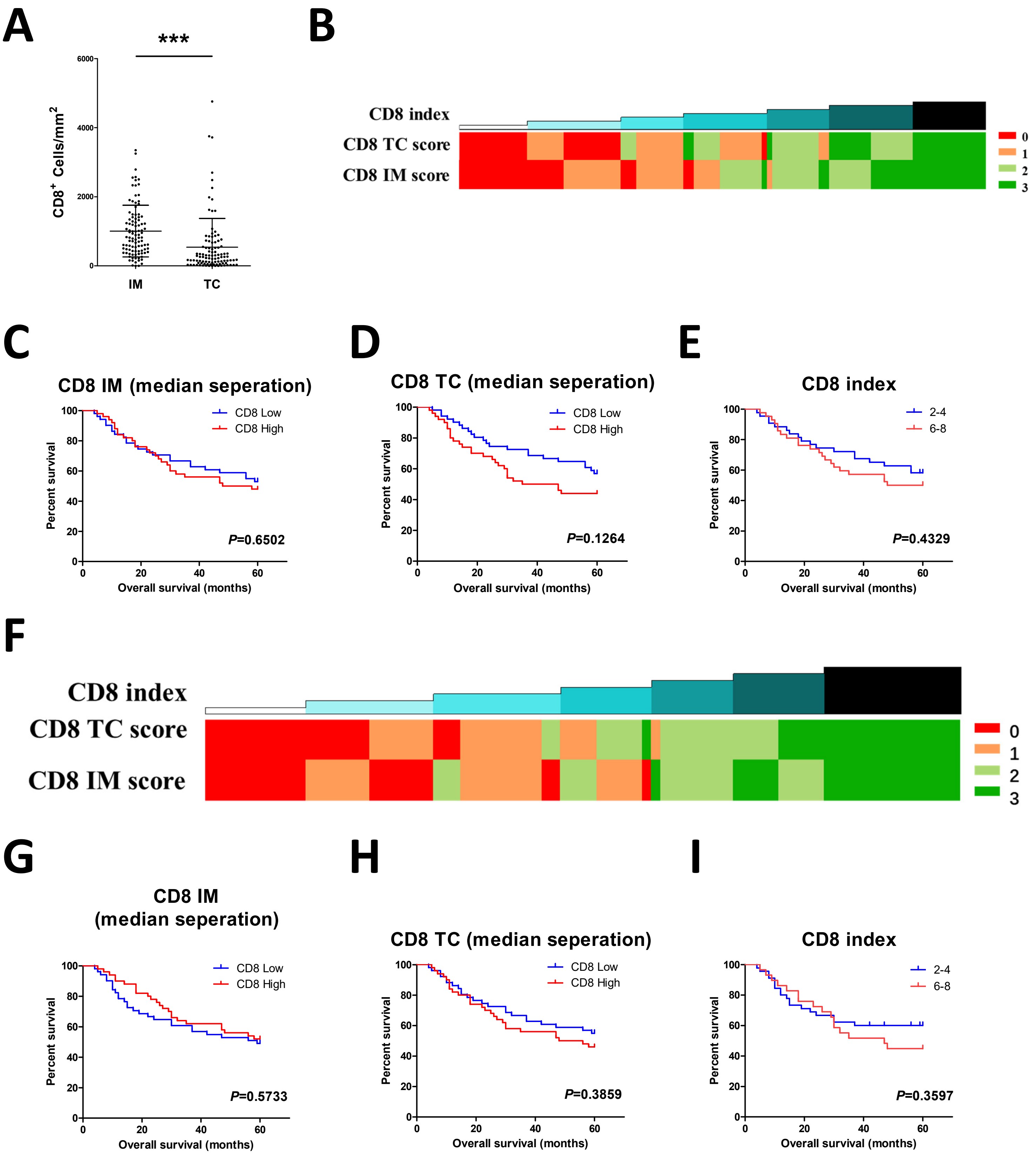

Supplementary Figure S1. No significant correlation was found between the CD8+ cell density and OS of 101 patients with HPV- HNSCC.

A, CD8+ cells Densities were determined using Fiji platform, , n=101. Error bars, SD. ***, $P < 0.001$. **B** and **F**, The CD8 score in IM, TC or combined areas. **B–D and E–F**, Density of CD8+ cells in IM (**B**, **F**), TC (**C** and **G**) or IM, TC combined (**D** and **H**) showed no correlation with OS. Densities of CD8+ were determined using Fiji platform (**A–D**) or StrataQuest platform (**E–H**). Log-rank test was performed to determine significance.

Supplementary Figure S2

A

	No.
#	263
Location	
Oral Cavity	47
Tongue (Oral)	66
Tongue (Base)	12
Larynx	72
Buccal	8
Gingival	8
Floor of mouth	23
Tonsil	19
Others	8

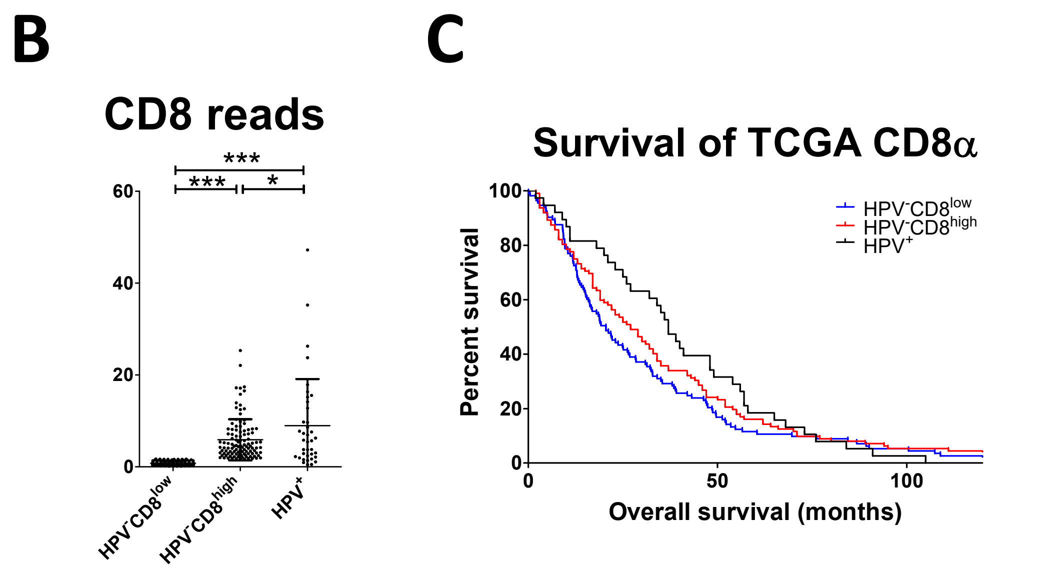

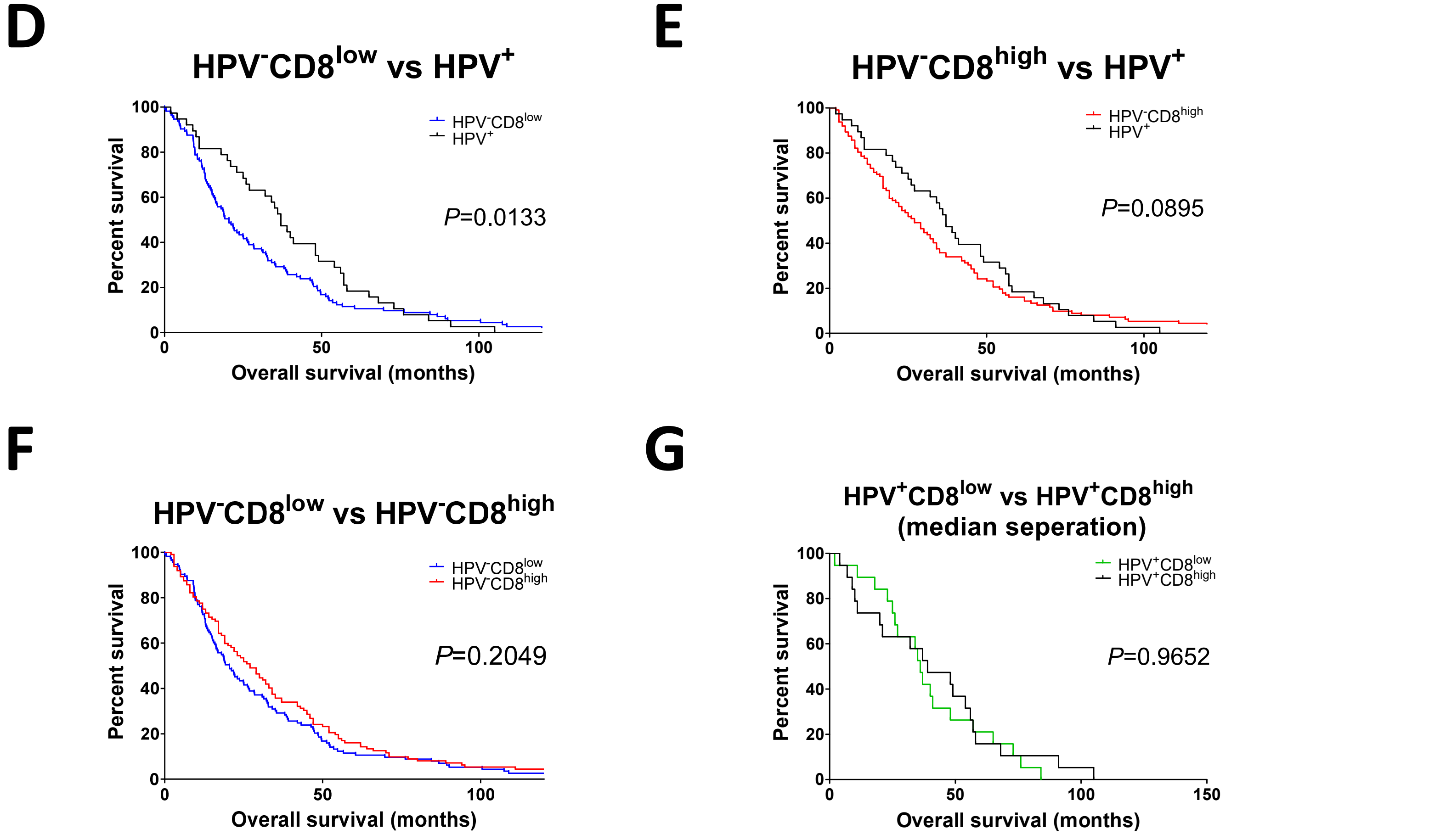

Supplementary Figure S2. No significant correlation was found between the $CD8^+$ cell density and OS in HPV^- HNSCC samples from TCGA.
A. Primary tumor site of 263 HNSCC. **B,** TCGA samples (n=263) were divided into three groups by CD8α expression: HPV^-CD8^{low}, HPV^-CD8^{high} and HPV^+. For HPV^- samples, those have CD8 value below the median is categorized as "$CD8^{low}$", otherwise "$CD8^{high}$". **C–G**. Kaplan-Meier analysis of OS of HNSCC patients classified as indicative subgroups. **D–G**, Gehan-Breslow-Wilcoxon test was performed to determine significance. Error bars, SD. *, $P < 0.05$; ***, $P < 0.001$.

Supplementary Figure S3

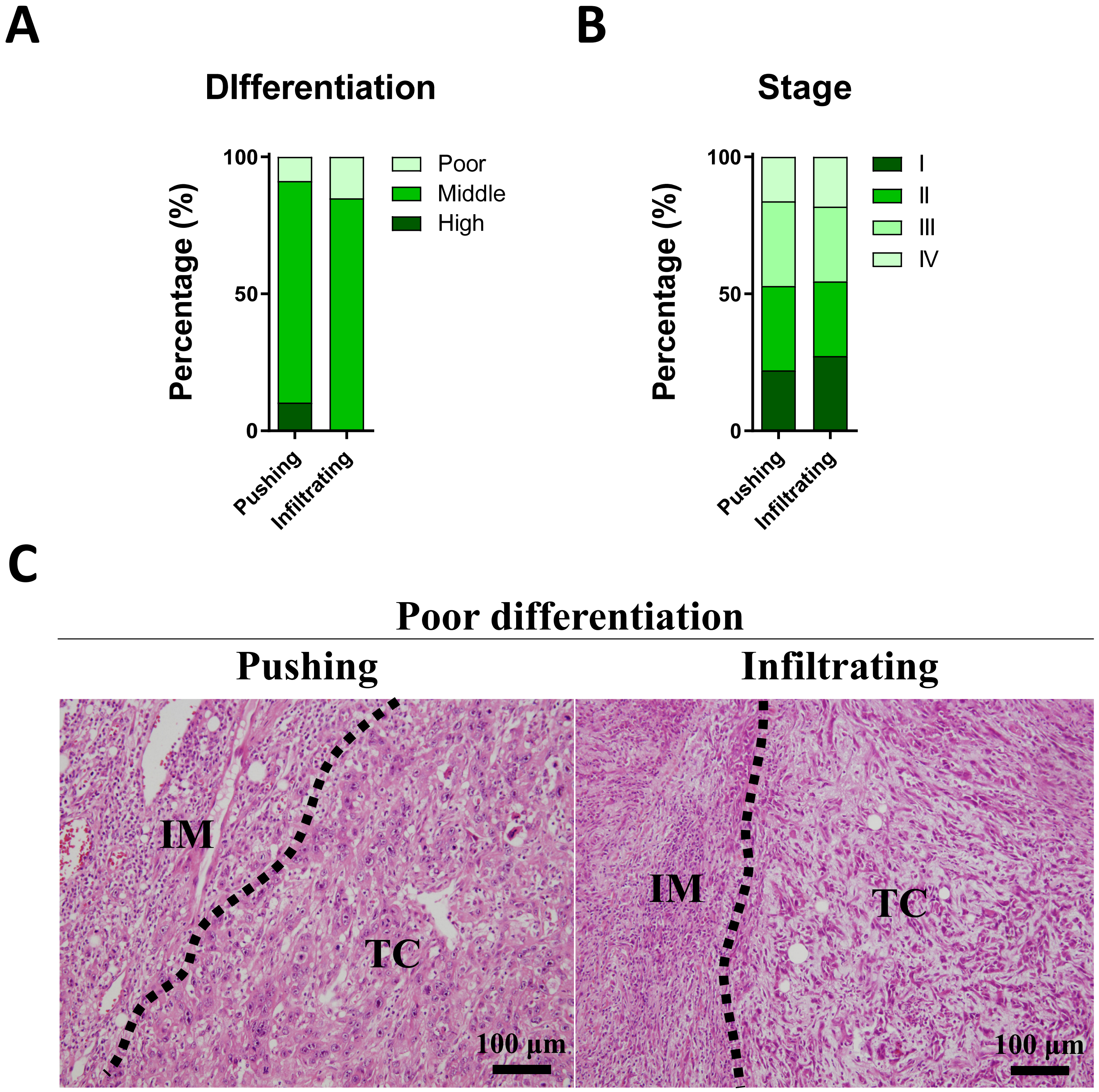

Supplementary Figure S3. Percentage of differentiation of tumor stage in indicated HPV- HNSCC subtypes. Stacked columns showing percentage of differentiation (**A**) or tumor stage (**B**) in HPV- HNSCC classified as "Pushing" (n = 68) or "Infiltrating" (n = 33). (**C**) Representative H&E images of poorly differentiated HNSCC. While well differentiated HNSCC cells resemble the benign squamous epithelium (large, polygonal, and sometimes with keratin pearl), poorly differentiated HNSCC cells have lost most of its squamous epithelial characteristics.

Supplementary Figure S4

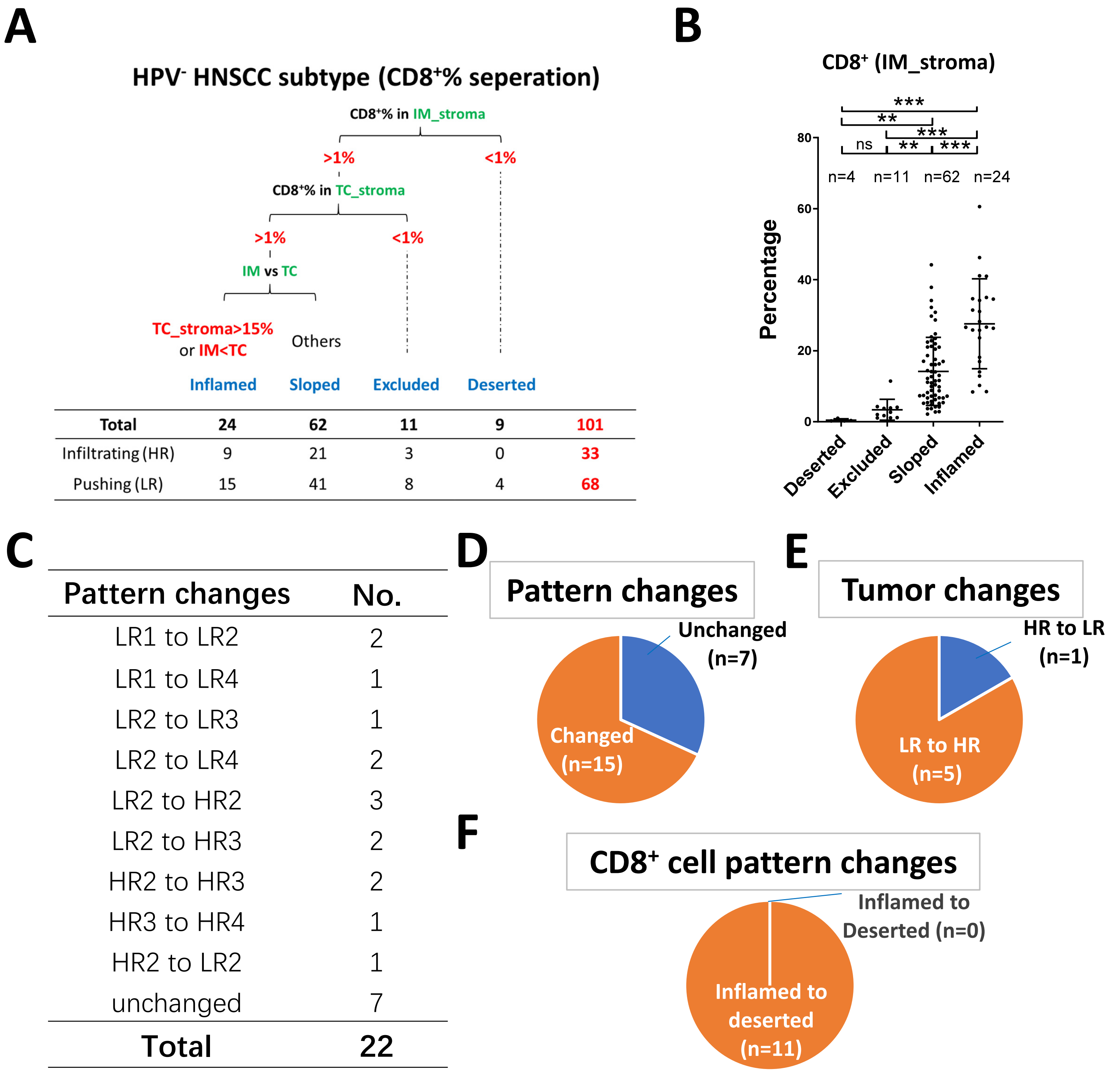

Supplementary Figure S4. Changes of tumor and CD8$^+$ cell pattern from primary to locally recurrent cancer. **A**, Criteria that samples were classified into four subtypes: inflamed, sloped (CD8$^+$%: IM_stroma and TC _stroma > 1%; IM>TC and TC _stroma <15%), excluded and deserted. **B**, Percentage of CD8$^+$ in IM _stroma of samples per group analyzed by StrataQuest. **C**. Classification and enumeration of different pattern changes. "LR" stands for Pushing (low risk); "HR" for Infiltrating (high risk); 1, 2, 3, 4 for inflamed, slope, excluded and deserted. For example, LR1 stands for Pushing (low risk) & inflamed tumor. **D–F**, Pie charts represent percentage of indicated pattern changes: total pattern changes (**D**), tumor changes (**E**) and CD8$^+$ cell pattern changes (**F**). **B**, Data are shown as mean with SD. **, $P < 0.01$; ***$P < 0.001$; ns, not significant.

Supplementary Figure S5

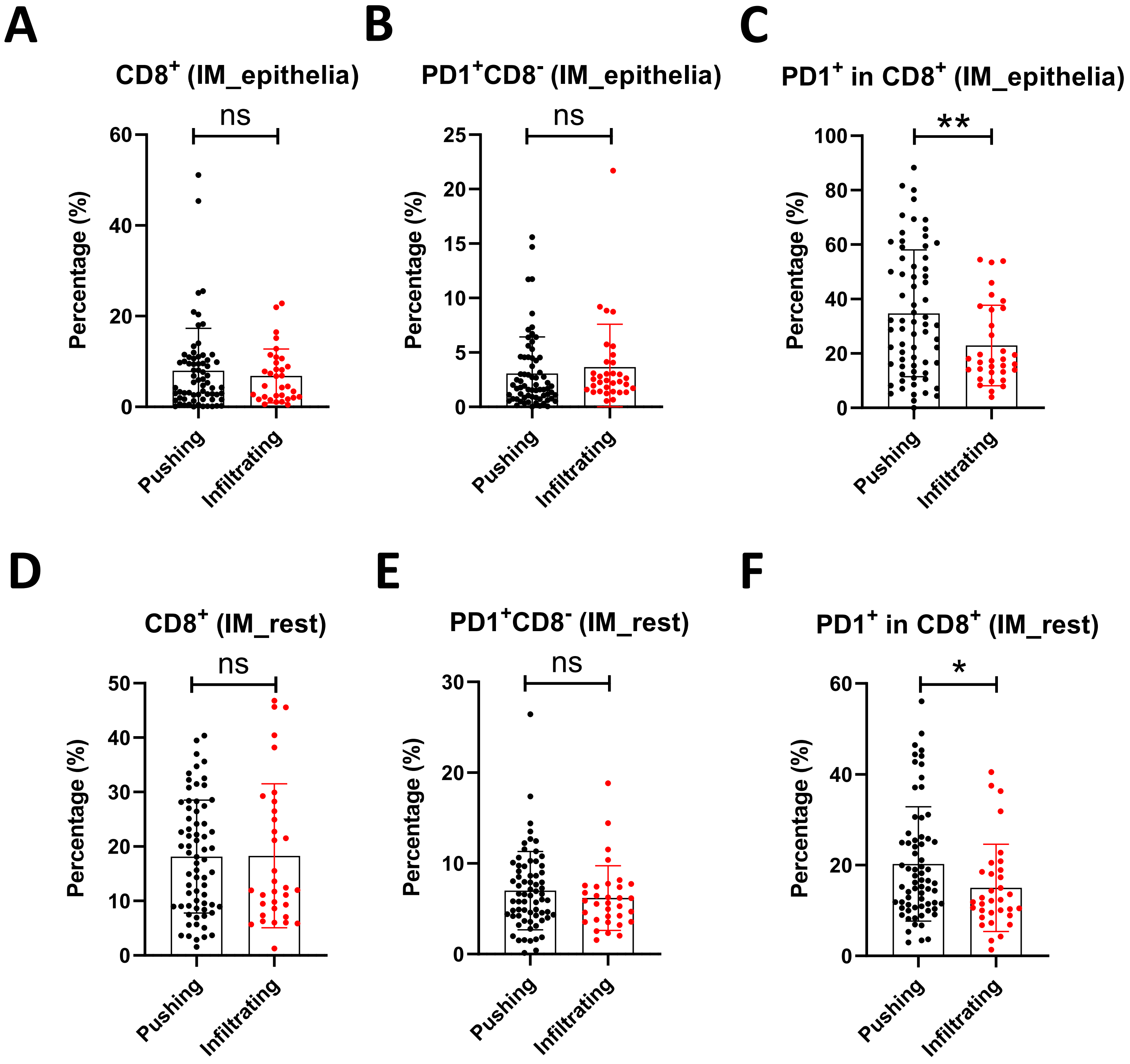

Supplementary Figure S5. Percentages of PD-1^{+}CD8^{+} TILs in different subareas of different HNSCC subtypes. Percentages of indicated cells in IM_epithelia (**A - C**) and IM_rest (**D - E**) were compared between “Pushing” (n=68) and “Infiltrating” HPV-HNSCC (n=33). **A - B**, data analyzed by StrataQuest are shown as mean with SD. *, $P < 0.05$; **, $P < 0.01$; ns, not significant.

Supplementary Figure S6

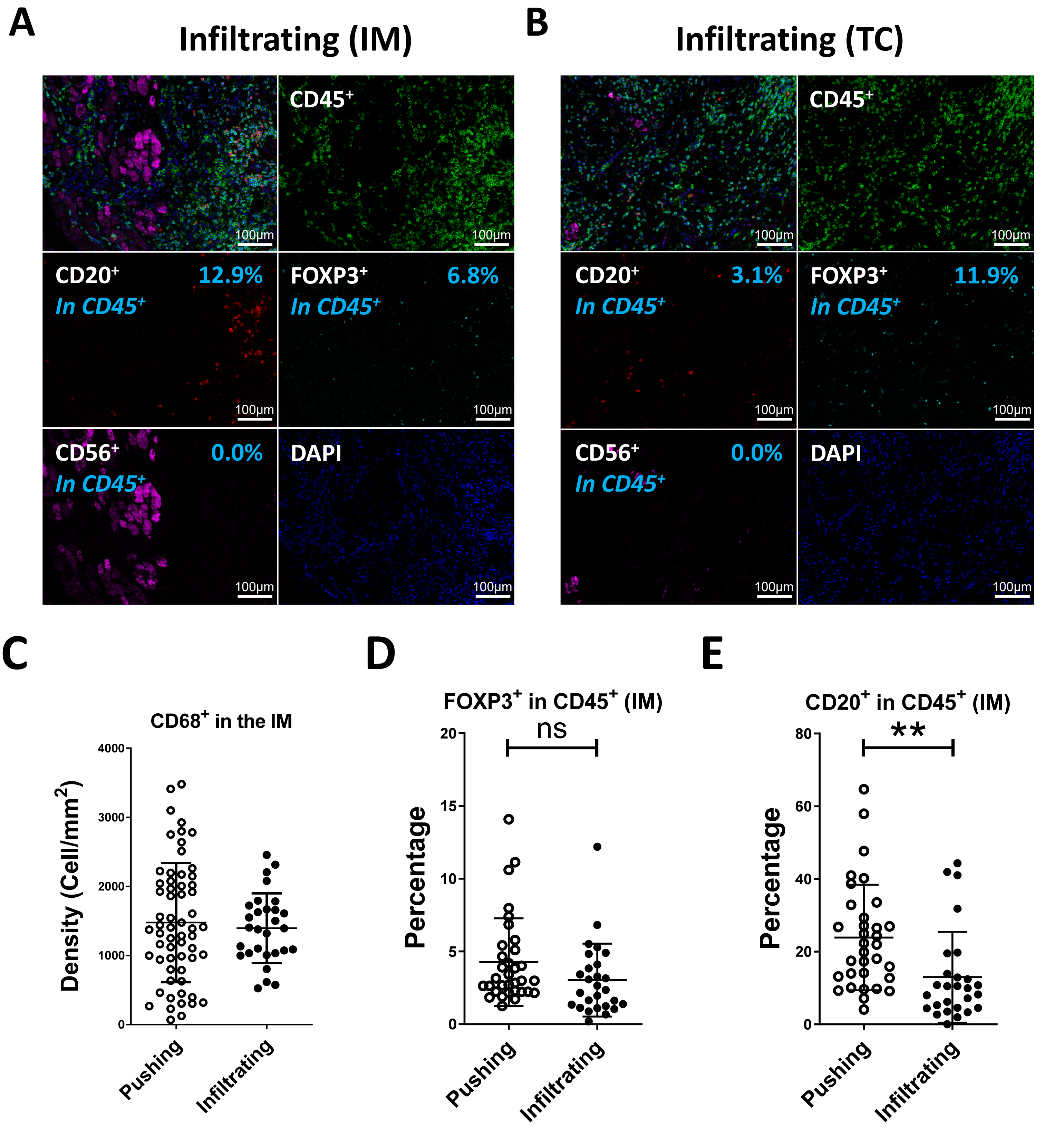

Supplementary Figure S6. Representative images of HNSCC stained with markers to identify different TILs. Panel consisting of CD45, CD20, CD56 and FOXP3 (order of antibodies: CD45, CD56, CD20 and FOXP3). Images were shown for IM (**A**) and TC (**B**). Percentages were over $CD45^+$ cells. (**C**) Densities of $CD68^+$ cells in indicated areas were analyzed by TissueQuest (n=101). (**D** and **E**), Percentages of indicated cells (out of $CD45^+$ TILs) in IM (analyzed by InForm) were compared between "Pushing" (n=33) and "Infiltrating" HPV^- HNSCC (n=27). Data are shown as mean with SD. **, $P < 0.01$; ns, not significant.

Supplementary Figure S7

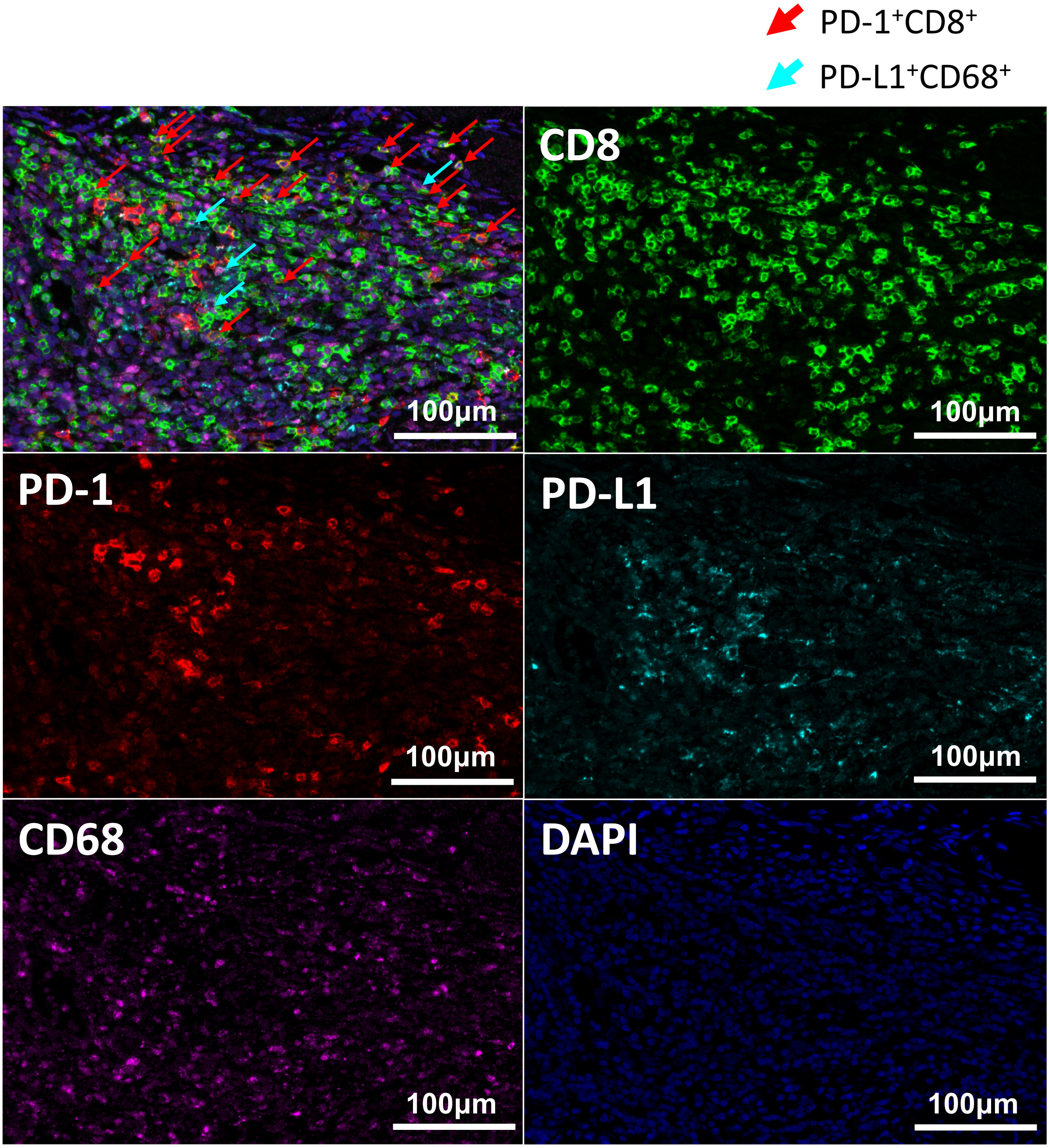

Supplementary Figure S7. Representative images of colocalization of PD-1^{+}CD8^{+} TILs and PD-L1^{+}CD68^{+} cells. PD-1^{+}CD8^{+} and PD-L1^{+}CD68^{+} cells were indicated by red and cyan arrows respectively.

Supplementary Figure S8

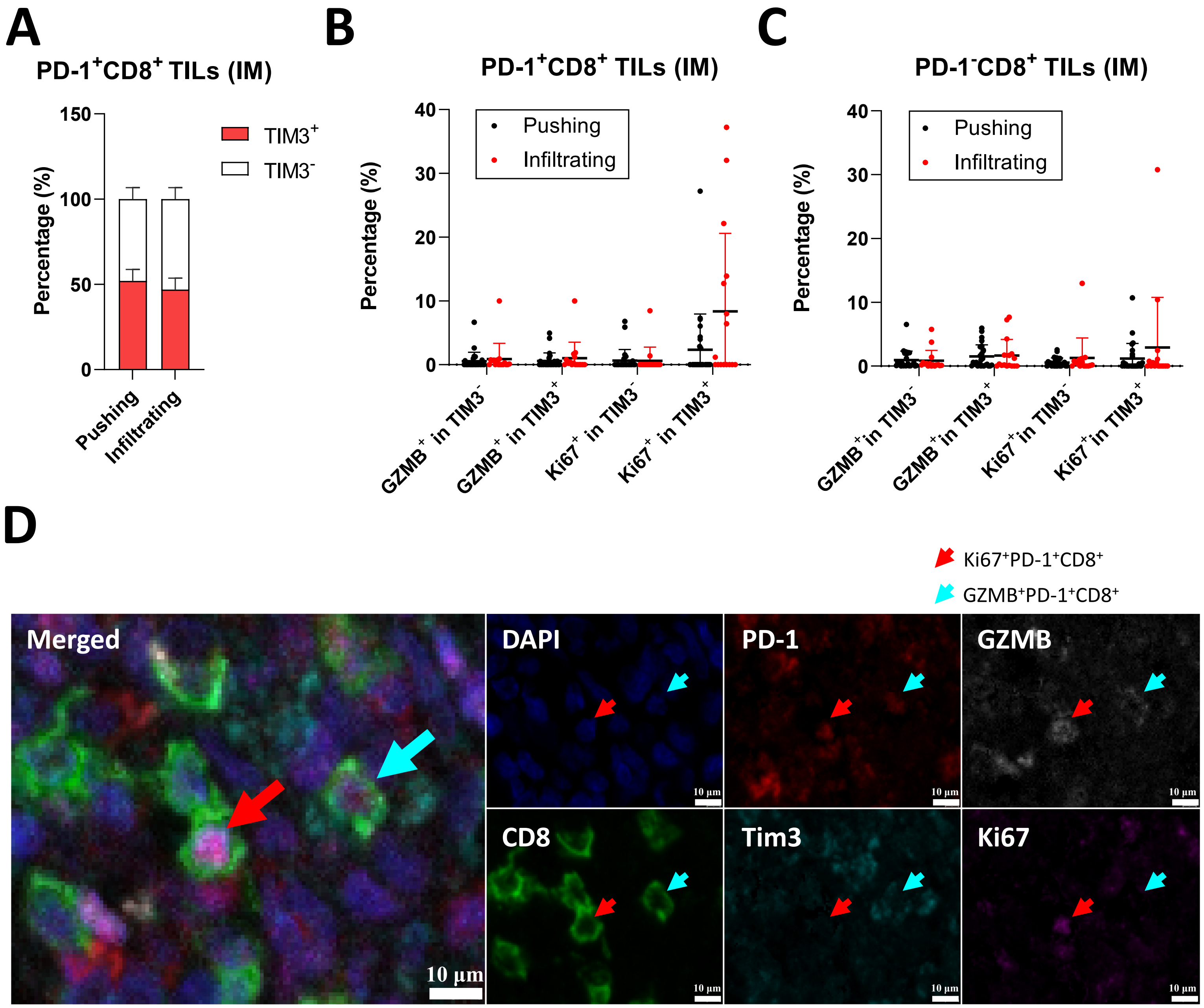

Supplementary Figure S8. Functionality of PD-1$^+$CD8$^+$ TILs in HPV$^-$ HNSCC. (**A**) Stacked columns showing percentage of Tim3$^+$- and Tim3$^-$- PD-1$^+$CD8$^+$ TILs in IM of "Pushing" (n = 26) or "Infiltrating" (n = 16) HPV$^-$ HNSCC. Data are shown as mean with SEM. (**B and C**) Percentages of GZMB$^+$ or Ki67$^+$ TILs in indicated subsets of PD-1$^+$CD8$^+$ (**B**) or PD-1$^-$CD8$^+$ TILs (**C**) in IM were compared between "Pushing" (n=26) and "Infiltrating" HPV$^-$ HNSCC (n=16). Data analyzed by TissueQuest are shown as mean with SD. (**D**) Representative images of GZMB$^+$PD-1$^+$CD8$^+$ TILs and Ki67$^+$ PD-1$^+$CD8$^+$ TILs in IM of HPV$^-$ HNSCC. Ki67$^+$PD-1$^+$CD8$^+$ and GZMB$^+$PD-1$^+$CD8$^+$ cells were indicated by red and cyan arrows respectively.

Supplementary Table S1. Baseline characteristics of 101 HPV- HNSCC.

	No.
#	101
Age	
<60	56
≥60	45
Gender	
male	68
female	33
Location	
Tongue (Oral)	71
Tongue (Oral)	65
Tongue (Base)	6*
Buccal	9
Gingival	9
Floor of mouth	6
Others	6
Grade	
I	7
II	83
III	11
Stage	
I – II	54
III – IV	47

* Tongue (Base): 6 of 71 (8.4%) tongue samples, 5 of 42 (11.9%) “Pushing” tumors and 1 of 29 (3.4%) “Infiltrating” tumors. No association between the two variables (HNSCC subtype and subsite) was found using chi-square test.

Supplementary Table S2. Information for pattern changes from matched primary to locally recurrent HPV⁻ HNSCC.

Primary				Recurrent				Treatment during transition	Pattern changes
**Number**	Surgery date	Nest	CD8 pattern	Number	Surgery date	Nest	CD8 pattern		
1	2011.1	HR	excluded	1	2011.11	HR	deserted	Radio	HR3-HR4
2	2011.06	LR	inflamed	2	2011.11	LR	deserted	No	LR1-LR4
3	2008.12	LR	sloped	3	2009.04	LR	deserted	Radio	LR2-LR4
				4	2009.06	LR	deserted	Radio	LR2-LR4
4	2011.09	HR	sloped	5	2011.11	HR	excluded	Chemo	HR2-HR3
5	2011.11	HR	sloped	6	2012.11	HR	excluded	No	HR2-HR3
6	2008.04	LR	sloped	7	2013.06	HR	excluded	Radio	LR2-HR3
				8	2015.05	HR	excluded	Radio	LR2-HR3
7	2007.1	LR	inflamed	9	2008.07	LR	inflamed	No	Unchanged
8	2011.08	HR	inflamed	10	2011.11	HR	inflamed	No	Unchanged
9	2008.12	LR	inflamed	11	2014.01	LR	sloped	Radio&Chemo	LR1-LR2
10	2007.12	LR	sloped	12	2008.06	HR	sloped	No	LR2-HR2
11	2008.06	LR	sloped	13	2009.06	LR	sloped	No	Unchanged
12	2008.09	HR	sloped	14	2012.01	LR	sloped	No	HR2-LR2
13	2008.09	HR	sloped	15	2011.06	HR	sloped	Radio	Unchanged
14	2009.02	HR	sloped	16	2009.11	HR	sloped	Radio	Unchanged
15	2009.05	LR	sloped	17	2012.08	LR	sloped	Radio	Unchanged
16	2010.06	LR	sloped	18	2012.08	HR	sloped	No	LR2-HR2
17	2010.09	LR	inflamed	19	2011.1	LR	sloped	Chemo	LR1-LR2
18	2009.01	LR	sloped	20	2009.11	LR	sloped	Radio&Chemo	Unchanged
				21	2009.11	LR	excluded	Radio&Chemo	LR2-LR3
				22	2009.11	HR	sloped	Radio&Chemo	LR2-HR2

"HR" stands for Infiltrating (high risk); "LR" stands for Pushing (low risk); 1, 2, 3, 4 stands for inflamed, slope, excluded and deserted respectively. Radio, radiotherapy. Chemo, chemotherapy. No, no radiotherapy and chemotherapy.

Supplementary Table S3. Baseline characteristics of 11 HPV^{+} HNSCC.

	No.
#	11
Age	
<60	3
≥60	8
Gender	
male	9
female	2
Location	
Tongue (Oral)	5
Tongue (Base)	3
Tongue (Oral)	2
Buccal	4
Gingival	1
Floor of mouth	1
Stage	
I – II	8
III – IV	3

Supplementary Methods

Immunohistochemistry

Multiplex IHC staining was performed as previously described with minor changes. Briefly, FFPE tissue sections of 3 μm thickness were deparaffinized and dehydrated, and antigens were retrieved (in citrate buffer (pH 6.0) using microwave heating). Detection of biomarkers was achieved by a multiplex IHC method using the Opal Kit (PerkinElmer).

Antibodies

Primary antibodies for IHC included P16INK4A (EPR1473; Abcam), CKpan (C11; Abcam), CD3 (SP7; Abcam), CD8 (C8/144B and D4W2Z; Abcam), PD-1 (D4W2J or EH33; CST and EPR20665; Abcam), CD4 (EPR6855; Abcam), CD14 (D7A2T; CST), CD68 (D4B9C; CST), CD45 (D9M8I; CST), CD20 (L26; Thermo Fisher Scientific), PD-L1 (MIH6; Abcam),B2m (EPR21752-214; Abcam) and Foxp3 (D2W8E; CST). Fluorescently conjugated antibodies were purchased from BD Biosciences (CD4-FITC, RPA-T4; CD4-Percpcy5.5, RM4-5; CD56-PE-cy7, B159; 4-1BB-PE, 1AH2; PD-1-PE/APC, J43; PD-L1-APC, MIH5; CD8-PE, RPA-T8) , Biolegend (H-2K^d/H-2D^d-PE, 34-1-2S; H-2K^k-PE, 36-7-5; β2-microglobulin-APC, A16041A; CD45-FITC, 30-F11; NKp46-PE, 29A1.4; CD3-APC, UCHT1; CD8-FITC and APC, 53-6.7; CD19-APC-CY7, H1B19; CD11b-Percp-cy5.5, ICRF44; CD14-PE, M4E2; PD-1-APC, EH12) and eBioscience (Foxp3-PE/Cy7, FJK-16s). PI, 7AAD and Zombie UV were from Biolegend. Therapeutic in vivo mAbs α–PD-1 (RMP1-14) and IgG isotype controls (2A3) were purchased from BioXCell.

PBMC and tumor samples

Blood and tumor specimens were collected at the beginning of the treatment. Peripheral blood mononuclear cells were isolated from centrifugation of whole blood using the Ficoll-Paque density gradient (GE Healthcare, Chicago, IL, USA). Fresh tissue from primary tumors was processed immediately after surgical resection and manually minced under sterile conditions followed by enzymatic digestion using RPMI-1640 (Thermo Fisher Scientific), 500 U/mL collagenase IV (Sigma), 2000 U/mL hyaluronidase, and 300 U/mL DNAse I (Sigma) at 37°C for 30 min. Then this suspension was filtered through 70 μm nylon cell strainers, and purified using density-based separation with Percoll (GE Healthcare).

Flow cytometry

At least 2×10^5 events per sample were acquired on FACS LSRFortessa (BD Biosciences) and $CD8^+$ T cell subsets were sorted using FACSAriaII instrument (BD Biosciences). Data were analyzed using FlowJo software v. 10.4.2 (Tree Star, Inc.).

Expansion of $CD8^+$ TILs from SCC7 tumor

Subcutaneously grown SCC7 tumor were processed as human tumor samples. The $CD8^+$ tumor-infiltrating lymphocytes were first isolated using CD8 (TIL) MicroBeads (Miltenyi Biotec) and then sorted by FACS. High purity of PD-$1^+$$CD8^+$ and PD-$1^-$$CD8^+$ TILs were expanded using complete T cell culture media (RPMI-1640 supplemented with 10% FBS, 1 mM sodium pyruvate, 2 mM l-glutamine, 2.5 mM HEPES, 100 U/ml penicillin, 100 μg/ml streptomycin and 3,000 U/ml rhIL-2 (Peprotech)).

4-1BB upregulation, IFN-γ release

After in vitro expansion of $CD8^+$ TILs, basal expression of 4-1BB was negative. For assessment of 4-1BB upregulation by TILs, $CD8^+$ TILs were cultured with target cells (1×10^5:1×10^5), in U-bottomed 96-well plates in complete T cell culture media without IL-2. Supernatants were harvested 24 hours later and analyzed for IFN-γ (Mouse IFN-gamma Valukine ELISA, Novus), and $CD8^+$ cells were analyzed for 4-1BB upregulation.

Cytolytic assays

Effector $CD8^+$ TILs and CFSE-labeled SCC7 cells were mixed at a range of E:T, cocultured in U-bottomed 96-well plates plating 1×10^4 targets/well and variable numbers of effector cells, with triplicate wells . Cultures were incubated for 24 hours at 37°C under 5% CO2. The 7-AAD was added to samples, and % cytolysis was calculated as 1 - % live cells. Specific lysisfor each sample was calculated as: $[(\%cytolysis_{sample} - \%cytolysis_{spotaneous}) / (\%cytolysis_{max} - \%cytolysis_{spotaneous})]$ and expressed as a percentage.

Cell lines and IFN-γ stimulation

The cell lines SCC7 and CT26 were originally obtained from American Type Culture Collection (ATCC), and cultured at 37 °C under 5% CO2 within RPMI-1640 culture medium supplemented with 10% fetal bovine serum (FBS) and 1% penicillin/streptomycin. For IFN-γ stimulation, tumor cells were seeded in 6-well plates (Corning) and cultured for 12 in growth medium, then growth medium containing 10 ng/mL IFN-γ (Life Technologies) was then substituted and cultured for an additional 48 h after which cells were analyzed by flow cytometry.
